# Ecologies of care: mental health and psychosocial support for war-affected youth in the U.S.

**DOI:** 10.1186/s13031-019-0233-x

**Published:** 2019-10-21

**Authors:** Cyril Bennouna, Maria Gandarilla Ocampo, Flora Cohen, Mashal Basir, Carine Allaf, Michael Wessells, Lindsay Stark

**Affiliations:** 1 Department of Political Science, Brown University, Providence, USA; 2 Brown School at Washington University in St. Louis, Missouri, USA; 3 Mailman School of Public Health, Columbia University, New York City, NY USA; 4 Qatar Foundation International, Washington, D.C, USA

**Keywords:** RefugeeMental health and psychosocial supportYouthAcculturationEducation

## Abstract

**Background:**

Youth resettling to the U.S. from conflict-affected countries in the Middle East and North Africa (MENA) face countless challenges. As they cope with their experiences of armed conflict and forced migration, these girls and boys must also adjust to the language and social norms of their new society, often encountering prejudice and discrimination along the way. Previous studies indicate that schools can play a central role in facilitating this adjustment while also promoting mental health and psychosocial wellbeing. This qualitative study aims to understand the lived experiences of MENA newcomers resettled in Austin, Texas and Harrisonburg, Virginia and to assess how schools, families, and communities support their mental and psychosocial wellbeing.

**Methods:**

We held six focus group discussions across the two cities with a total of 30 youths (13–23 years) from Iraq, Syria, and Sudan. We also conducted semi-structured interviews with 30 caregivers and 27 key informants, including teachers, administrators, service providers, and personnel from community-based organizations.

**Results:**

Guided by Bioecological Theory, our thematic analysis identifies several means by which various actors work together to support resettled adolescents. We highlight promising efforts that seek to enhance these supports, including sheltered instruction, school-parent collaboration, peer support programming, social and emotional learning initiatives, and integrated mental health centers.

**Conclusion:**

While this study underscores the resilience of newcomers and the value of local support systems, it also reflects the importance of investment in schools, mental health systems, and resettlement programs that can enable newcomers to achieve their full potential.

## Introduction

The United States has a long history of welcoming immigrants fleeing armed conflict and persecution. From 1975 to 2018, the U.S. Refugee Resettlement Program welcomed an estimated 3.4 million refugees around the country [1]. The Trump Administration, however, has substantially reduced the resettlement program in recent years, resettling 30,000 people in FY 2019, and planning to limit admissions further to 18,000 in FY 2020 [2]. Typically, two of every five refugees resettled to the U.S. is under 18 years old [3].

Resettled children and adolescents face a number of difficulties due to their pre-migration and displacement experiences, often including exposure to violence, family separation, and lost years of education, among other hardships. Many of these girls and boys struggle with psychological distress resulting from repeated exposure to adversities and the loss of their social supports [4]. As they cope with these adversities, resettled girls and boys may also struggle to learn the language and social norms of their new society [5]. When they begin reintegrating into schools, these newcomers have to compete with students who, for the most part, have spent the majority of their lives in American schools.

While parents struggle to support their children’s adjustment, they also face their own difficulties, including finding and keeping gainful employment, navigating access to transportation and public services, and managing households [6, 7]. Even those with higher educational achievement and skills training in their home countries tend to struggle to find well-paying jobs after resettlement, as U.S. employers often do not recognize their advanced degrees or employment credentials [8]. Since parents work long hours, and children typically pick up conversational skills more quickly than their parents, many family responsibilities fall on older children [9].

Nevertheless, a sizeable proportion of girls and boys resettled to the U.S. overcome these challenges, often through a variety of protective and promotive factors that foster resilience [10]. These protective factors span the individual, family, community, and societal levels. A range of mental health and psychosocial support (MHPSS) programs aim to help newcomers by preventing suffering, reinforcing strengths, promoting psychosocial wellbeing, and treating mental disorders [11]. In principle, MHPSS systems should consist of four layers (or “tiers”) of support, including: [1] the universal provision of basic services in a safe, participatory, and socially responsible manner that promotes mental health and psychosocial wellbeing [2]; strengthening family and community supports [3]; focused services from non-specialized workers (e.g. psychosocial first aid); and [4] specialized support from mental health professionals (e.g. Trauma-Focused CBT) [11, 12].

Schools play a central role in supporting young people’s adjustment to their new homes through a variety of academic, social, and emotional supports [5, 13]. School-led social and emotional learning (SEL) initiatives focus on helping youth “develop the ability to recognize and manage emotions, develop caring and concern for others, make responsible decisions, establish positive relationships, and handle challenging situations effectively” [14]. More broadly, schools can deliver multi-layered MHPSS directly to students or connect students and families to appropriate services. A growing literature has documented that school-based SEL and other multi-layered MHPSS activities can promote resilience among resettled children and adolescents, but more research is needed to understand how schools, and school systems, work with students, families, and communities to deliver such supports [15–17].

According to data from the U.S. State Department, which classifies refugees according to seven geographic regions, individuals from what it titles the Near East and South Asia constituted the single largest regional group of refugees admitted to the U.S. between 2008 and 2017, ranging from 35.1 to 51.6% of all admissions [18]. Although the number and proportion of refugees from this region have diminished substantially in recent years under the influence of the Trump Administration’s travel bans and new admissions ceilings, 33.6% of arriving refugees in 2018 came from the Near East and South Asia region [18]. Despite constituting such a large proportion of newly arrived refugees in the U.S. over the past decade, the experiences, needs, and capacities of students resettled to the U.S. from the MENA region have been largely overlooked. Girls and boys from countries such as Syria and Iraq may have unique experiences with resettlement in the U.S. owing in part to high levels of discrimination against individuals from the Middle East and North African (MENA)^1^ region in the years following the terrorist attacks of September 11, 2001 and the subsequent Global War on Terror [19, 20]. Anti-MENA sentiment perpetuated by mass media and policymakers has contributed to a climate of overt prejudice and discrimination against MENA-identified individuals in the U.S. [19]. In 2017, for example, anti-iIslamic offenses comprised 18.7% of all religion-based hate crimes, a steep increase from 2% before September 11, 2001 [21, 22]. Furthermore, Muslims report higher perceived discrimination than their Christian counterparts [23].

Additionally, individuals from the MENA region may face acculturation stressors regarding the dominant attitudes, behaviors, and social norms of their new societies. For example, whereas traditional gender roles may regard women as caregivers more than breadwinners, women often need to work upon their arrival to the U.S., potentially shifting parental and spousal relationships. While parenting styles differ across the MENA region, parents accustomed to more authoritarian parenting styles, and the use of corporal punishment, may have difficulty adjusting to environments promoting more permissive or flexible parenting styles [24, 25]. What constitutes “health” can also vary across newcomer groups, especially regarding mental health and psychosocial wellbeing, which can be highly stigmatized. Differences of interpretation related to defining, identifying, and responding to idioms of distress complicate effort to communicate about MHPSS transculturally and can lead to underutilization of available services [25].

### Study objectives

This study aims to understand the lived experiences of MENA newcomers resettled in Austin, Texas and Harrisonburg, Virginia. In particular, we intend to:

1. Identify common challenges that adolescent newcomers from the MENA region, and their families, encounter regarding acculturative stress and psychosocial adjustment; and
2. Assess how individuals, schools, families, and communities currently support the mental and psychosocial wellbeing of adolescent newcomers from the MENA region, including the perceived strengths and limitations of these supports;

By documenting the experiences of this often-neglected sub-population and locating the possible mechanisms through which they adapt to their new schools and communities, we hope to inform future research efforts to quantify the MHPSS needs and capacities of conflict-affected newcomers from the MENA region. This study should also provide guidance for the development of programs and policies aiming to strengthen the environment of care for these newcomers.

### Conceptual framework

**Fig. 1:**
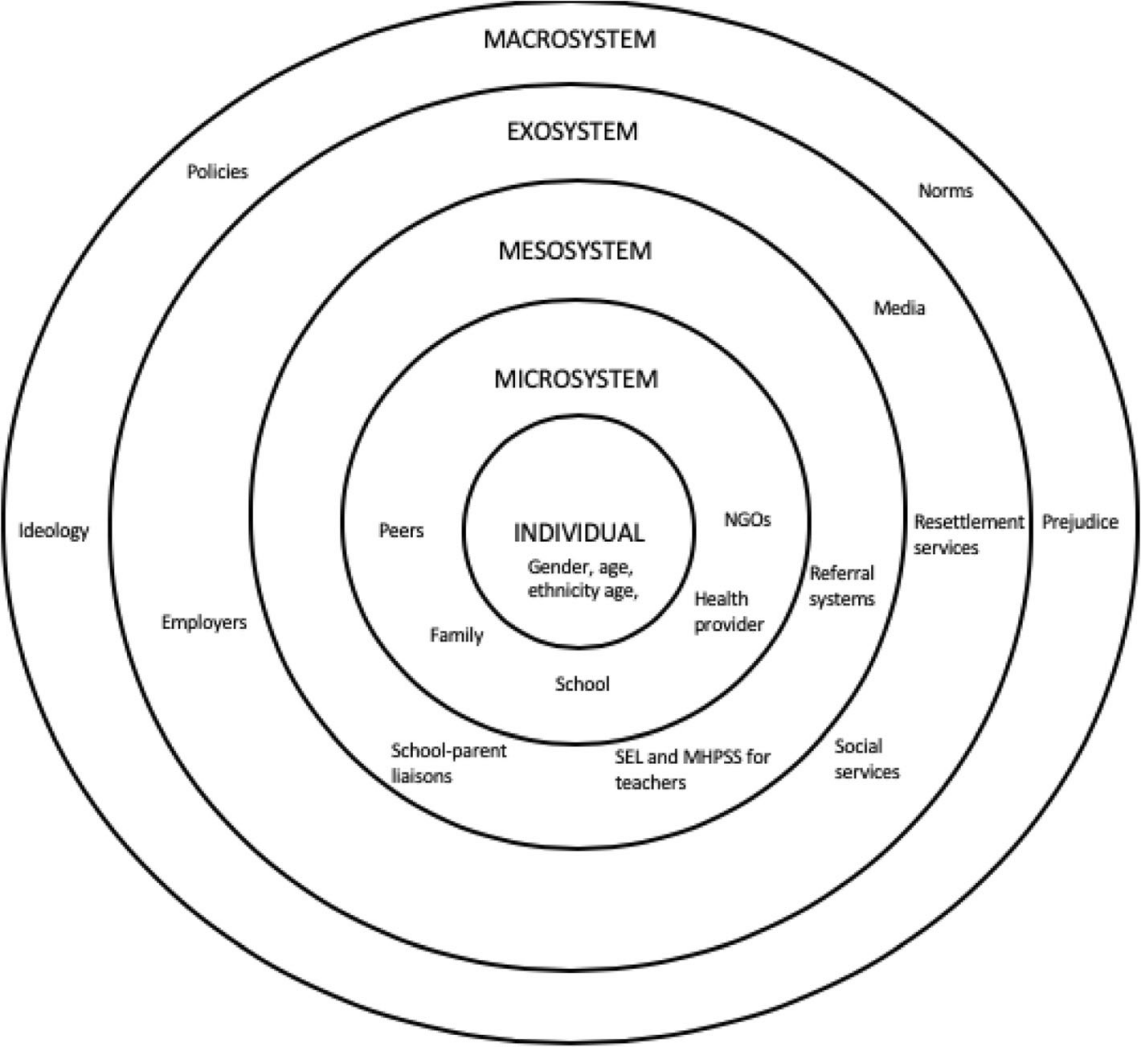
Adapted Illustration of the Bioecological Theory (Bronfenbrenner, 1979; Bronfenbrenner & Morris, 2006)

The Bioecological Theory of Human Development provides a useful starting point for studying how the different supports in an adolescent’s life influence their outcomes after resettlement [26, 27]. The theory holds that human development takes place through processes of interaction between the individual and the “persons, objects, and symbols in its immediate external environment” [27]. These processes, which can include for instance studying, problem solving, and caring for others, are in turn influenced by the characteristics of the person and the context and time in which they live. Scholars and practitioners working with populations affected by armed conflict have usually focused on the ways in which contextual factors at various ecological levels influence young people’s outcomes (Fig. 1). Factors in an adolescent’s *microsystem*, or immediate environment, include their interactions with family members, peers, educators, and service providers. The relationships between these different microsystems—for instance, how family members interact with the school—constitute the individual’s *mesosystem.* Factors in an adolescent’s *exosystem*, meanwhile, influence the adolescent indirectly, such as a parent’s relationship with their workplace. Finally, the *macrosystem* includes factors at the broadest levels of society, such as policies, political ideology, prejudice, and social norms, which influence adolescents through the micro-, meso-, and exosystems.

Scholars have adapted the Bioecological Theory in various ways to study resilience [28, 29], acculturation [30], and ethnic trauma [19], and to assess the educational needs of resettled refugees [31, 32]. In line with several of these authors, we distinguish between pre-migration, transmigration, and post-migration social ecologies [31]. This change of social ecological context is the defining feature of migration, and although it may confer a range of benefits both to the individual and the welcoming society over time, the acculturative stress that accompanies migration and resettlement may also compromise an adolescent’s mental health and psychosocial wellbeing [19, 33, 34]. The context of reception in the receiving community, including its degree of openness, provision of support, acceptance of newcomers, and opportunity structures, can mitigate or exacerbate this acculturative stress [35].

## Methods

### Setting

Data collection took place in Austin, Texas and Harrisonburg, Virginia. We selected these sites purposively to reflect two cities of varying sizes with a relatively high number of families from conflict-affected MENA countries. In order to provide a setting where participants could engage with the research comfortably and openly, moreover, we selected two districts/divisions where the study sponsor, Qatar Foundation International (QFI), had strong partnerships and where school leadership expressed an interest in using research findings to inform future programming efforts.

According to 2017 estimates, Harrisonburg has a population of 54,215, compared to Austin’s 950,715 [36]. While updated estimates are not available by city, Harrisonburg and Austin have historically been leading sites of resettlement in their states. Virginia welcomed some 14,672 (2.1%) of the 697,862 refugees resettled in the U.S. since 2008, while Texas welcomed 69,832 (10%) [2]. In 2015, some 28.1% of refugees and special immigrant visa (SIV) holders resettled in Virginia were from the MENA region, compared to 19.5% in Texas and 15.6% nationally [37]. Iraqis represent by far the largest national group resettled from the MENA region. Since 2008, Virginia has resettled some 5450 Iraqis and Texas resettled 18,915 [38].

Harrisonburg has often been considered an exceptionally hospitable place for immigrants in Virginia [39]. In 2016, the City Council passed a resolution declaring Harrisonburg a “Welcoming America” city, making it part of a national network of communities working together to improve the context of reception for immigrants and refugees [40]. Church World Service (CWS) manages refugee resettlement in Harrisonburg, connecting recent arrivals to housing, basic services, education, and employment. The rate of refugee resettlement had fallen sharply at the time of data collection, especially among families from the MENA region, owing to the new ceiling on arrivals imposed by the Trump Administration as well as the travel ban [41]. Harrisonburg City Public Schools (HCPS) serves some 6000 students, of whom a little under half (46%) were born outside the U.S. After Spanish, Arabic (9% of students) and Kurdish (6% of students) are the most common foreign languages [42].

Austin has been a central example of American cities struggling with state-led efforts to reduce immigration and refugee resettlement in recent years. In 2016, for example, the State of Texas formally withdrew from the federal Refugee Resettlement Program, leaving cities and their networks of nongovernmental organizations (NGOs) to support incoming refugees alone [43]. Until 2018, Refugee Services of Texas (RST) and Caritas of Austin worked together to serve newcomers, but the latter organization ended its resettlement program in 2018, owing to a drastic reduction in refugee arrivals [44]. Austin Independent School District (AISD) serves some 81,650 students, of whom some 27% are English language learners (ELLs) [45]. After Spanish, Arabic is the most common home language, and there were some 75 Arabic-speaking students from conflict-affected countries enrolled in 13 of AISD’s 14 high schools in 2018, according to internal data.

### Participants

Three groups of participants were eligible for participation in this study, including: 1) youth enrolled in high school (13–23 years) that were either born in conflict-affected MENA countries or born during their family’s displacement from such a country; 2) the caregivers of these youths, including parents and siblings; and 3) key informants responsible for services, programming, or policy related to the first two groups. Key informants included teachers, guidance counselors, school district/division administrators, case workers, therapists, and NGO personnel.

In Harrisonburg, the study team worked with a division official to develop a list of potential key informants to recruit purposively. The administrator helped recruit and schedule school key informants, while the study team contacted non-school key informants directly via email. All key informants provided written consent. The interviewers also asked each key informant to recommend other participants. For caregivers and youths, the study team worked with school personnel and a representative from CWS to develop a list of potential participants that had agreed to be contacted by the research team. The researcher and a trained Arabic interpreter then contacted adult caregivers by phone to explain the study purposes and to inquire about their interest in the study. Those who expressed interest were mailed a study information form in English and Arabic. The form also included the option to decline to be contacted further. Of the 25 families contacted, 17 participated, one declined, and the rest could not be reached. A researcher and interpreter then visited each family that expressed interest in order to present the study, answer questions, and complete written informed consent (including consent for adults to participate and consent to invite their youths to participate). Youths were then invited to the public high school and they completed written informed assent in the absence of their parents in order to ensure their participation was voluntary.

In Austin, the study team worked with AISD’s Refugee Family Support Office to develop a list of potential key informants, caregivers, and youths. The research team contacted key informants directly by email or phone and all key informants provided written consent in person. Key informants were also asked to suggest other potential participants. An Arabic interpreter trained by the research team contacted all potential family participants and those who agreed were mailed adult participant and parental consent forms. Of the 48 families contacted, 14 participated, eight refused, five consented but did not show up for data collection, and the rest could not be reached. The research team and interpreter visited all families who expressed an interest in participating to complete written informed consent. Youths were invited to a public high school where they completed written informed assent.

### Data collection

The research team conducted a scoping study in Harrisonburg in February 2018 and in Austin in March 2018 in order to coordinate with school partners, identify potential participants, contextualize data collection instruments, and plan data collection. Data collection took place in both locations throughout July 2018 and consisted of semi-structured interviews with caregivers and key informants and school-based focus group discussions (FGD) with youths. In two-parent homes, parents were often interviewed together, though it was common for only the mother to be available. Key informant interviews sometimes included two participants, while FGDs, which were stratified by gender, included five students per session, on average.

Interview questions for caregivers focused on displacement and resettlement experiences, acculturative stressors, engagement with schools and healthcare, sources of support, and perceptions of children’s adjustment (Table 1). Interview questions for key informants were tailored to participants’ job responsibilities, but focused centrally on challenges faced by newcomers and the supports in place to reinforce student strengths and promote positive outcomes. Focus groups discussed the interactions that conflict-affected students from the MENA region had with educators, parents, and peers, and students. Students also provided their own definitions of what it meant to feel supported and proposed ideas for improving school- and community-based support systems. Caregiver and youth sessions were assisted by an Arabic interpreter trained in best ethical and methodological practices for interviewing youths affected by armed conflict. The study team did not ask about participants’ refugee or citizenship status. The researchers recorded all data collection sessions using an audio recording device, unless the participant requested otherwise, and also kept detailed field notes. A team transcribed the audio files, including the interpreter’s Arabic-to-English translation. The research team reviewed, edited, and de-identified all transcripts in English.

**Table 1:** Examples of Interview and FGD Questions

	Adolescent FGDs	Adult Caregiver Interviews	Adult Professional Interviews
1	How do students in this school make new friends? What are some places where you can meet new friends? What are some things that would make people not become friends? How do friends support one another?	What was your experience of arriving to the U.S. like? Please describe some of the ways you first felt. What kinds of support did your family receive from thegovernment or other organizations when you first arrived? What are some additional kinds of support that you receive from yourcommunity?	What would you say it is like for a refugee student of Arab background to attend school in this district? How do you think their experiences in the school compareto those of other students?
2	Who are some of the adults in theschool that you would go to if youwanted to speak about your future? What would you do if you were worried that you had a health issue? For instance, if you felt too tired or if you felt sad? Is there someone in the school you would speak with?	People have different views on mental health. What does mental health mean to you? Is mental health something that you speak about sometimes in your family?	What has your experience been working with the families of refugee youth from Arab backgrounds?
3	What does it mean for a school to support a student? What do schools do to make students feel safe? What do schools do to make students feel like they belong?	How would you describe your relationship with his/her school? For example, do you attend meetings at the school? Are you in touch with any teachers or counselors? What has contributed to this relationship being the way it is?	What has been the role of the school administration/service team/organization in supporting refugee students from Arab backgrounds to integrate into their new communities? How would you say these strategies are working? What would you change about these current strategies and why?

### Ethical considerations

Recognizing the ethical complexities of collecting data with populations affected by armed conflict and forced migration, and particularly adolescents, the research team took several measures to promote voluntary, meaningful, and safe participation [15, 46]. Such measures included: 1) partnering with schools and community NGOs that could provide safe research space, make referrals in case of acute needs, and protect participant confidentiality; 2) engaging with interpreters from the MENA region; 3) training everyone involved in data collection on ethical conduct; and 4) de-identifying and securing participant data. Furthermore, the research team considered informed consent and assent an ongoing process that required open communication and accessible, child-friendly research instruments [47].

The research protocol was approved by the Institutional Review Boards (IRB) at Columbia University’s Mailman School of Public Health (IRB-AAAR7830), AISD’s Department of Research and Evaluation (R18.62), and the Superintendent of Schools at HCPS.

### Analysis

The research team analyzed the data using thematic analysis and constant comparative method [48, 49]. The lead analyst reviewed all transcripts to develop initial codes related to the research objectives and questions. He then compared these codes to the relevant literature and developed analytic memos and an initial codebook. After incorporating feedback from the principal investigator and research team, the lead analyst then applied the codebook to a sample of the data and refined the codebook further. The researchers then recruited a team of coders with backgrounds in public health, social work, psychology, and refugee resettlement and trained them on the study protocol and codebook. Together, the research team finalized the codebook with 56 codes and built inter-coder reliability (ICR) using *Dedoose’s* Training Center (Additional file 1). Once coders had established an ICR of at least 66.7% on tested codes, they were assigned a batch of transcripts to code using *Dedoose*. The lead analyst reviewed coding applications and resolved ambiguities in coding application. Through an iterative process, the lead analyst and principal investigator identified eight themes in the data and compared these to the empirical and theoretical literatures related to Bioecological Theory, acculturation, adolescent SEL, and the mental health and psychosocial wellbeing of forced migrants (Additional file 1).

## Results

We conducted interviews with 27 key informants and 30 caregivers across the two cities, as well as six FGDs with 30 youths (Table 2). Key informants included eight teachers and high school principals, nine district/division staff, and 10 NGO personnel from resettlement organizations and community-based organizations. Altogether, the study engaged youths and caregivers from 31 households, the majority (68.3%) of whom came from Iraq, with a sizeable minority from Syria (18.3%) and Sudan (13.3%) (Table 3). It is important to note that within these national identities there was considerable ethnic and religious diversity. The Iraqi group, for example, included participants identifying as Kurds, Yazidis, and Turkmen. Families had complicated migration histories, often having spent years in refugee camps or settlements in countries such as Jordan, Turkey, Egypt, and Libya. At least four families had fled the Iraq war for Syria, only to encounter that country’s civil war. Ongoing displacement and uncertainty about their final destination left many families precarious during this period, sometimes interrupting children’s education for years at a time. Several children had been born in displacement, spoke local languages, and partially identified with their country of refuge. Participants frequently remained separated from their extended family members, and in at least one case, an immediate family member had been prevented from traveling to the U.S. following the Trump Administration’s various travel bans.

**Table 2:** Number of Participants by Study Group and Location

	Key informants	Caregivers	FGDs	Total
Harrisonburg				
Sessions	10	10	3	23
Total participants	10	14	17	41
*Females*	6	8	9	23
*Males*	4	6	8	18
Austin				
Sessions	15	10	3	28
Total participants	17	16	13	46
*Females*	12	9	3	24
*Males*	5	7	10	22
All sites				
Sessions	25	20	6	51
Total participants	27	30	30	87
*Females*	18	17	12	47
*Males*	9	13	18	40

**Note.** Two adolescents participated in both caregiver interviews and FGDs, so the total number of unique participants is 85.

**Table 3:** Countries of Origin for Adolescent and Caregiver Participants

	Austin		Harrisonburg		All sites	
	Participants	Percentage	Participants	Percentage	Participants	Percentage
Iraq	16	55.2	25	80.6	41	68.3
Syria	8	27.6	3	9.7	11	18.3
Sudan	5	17.2	3	9.7	8	13.3
Total	29	100	31	100	60	100

### Adjustment challenges

The families we interviewed had lived in the U.S. for anywhere between 2 and 9 years, with a median of 4 years. Adults usually worked long hours in manual and low-skilled jobs, such as food processing, construction, and transportation, despite often coming from more skilled professions in their countries of origin. Youths and especially adults described considerable difficulty learning English, which undermined their career opportunities and ability to engage with public services, such as healthcare and education. As one father explained:

[W] *ithout the language, everything will be difficult. My intention was that when I come to U.S., I work in the same field—in construction. But because my language wouldn’t help me, so I couldn’t work in the same field* (Austin, Father, Sudan, A2.008.1).

Although schools, resettlement agencies, and youths themselves helped caregivers communicate across languages, caregivers did not always understand or agree with the policies, practices, and pedagogical approaches of their children’s new schools. As one boy explained about his parents, for instance:

*They don’t know much about school, especially the systems and stuff. So, all, everything they want is like to ask that we are fine* [*…*] *but they really don’t know what’s exactly going on in the school* (Harrisonburg, Boy, Iraq, H3.016.1).

While most parents in both cities expressed largely positive feelings about their schools and communities, a number also shared concerns about the potential “bad” influence of other students on their children, what they perceived as a permissive disciplinary environment, and the use of technology such as computers for education. In Harrisonburg, for example, where students were given laptops to take home, parents sometimes felt that these laptops created a barrier between children and their parents, who could not always monitor or participate in computer-based educational exercises. Meanwhile, parents in Austin were sometimes confused that their children did not bring home textbooks. As one parent explained:

*I don’t understand. What’s the idea of keeping these books in the school, not bringing them home with the students so at least the parent can have knowledge and* [*…*] *at least have an idea of what their children are studying* (Austin, Mother, Iraq, A2.011.1)?

For students, the most salient challenge, beyond language acquisition, was learning to navigate the school system. Most students described an initial sense of disorientation when they began school, not only because of their English level, but also because they were not accustomed to multiple class periods, were not aware of the full range of programs and extra-curricular activities on offer, and did not have enough background contextual information to follow their courses. As a male student from Iraq explained about his History teacher in Harrisonburg: “He would say some stuff that a U.S. person would know, but I had no idea what they were and he expected everyone to already know that part, so he would jump from place to place” (H3.016.1).

Beyond academics, students in both sites described experiences with bullying and microaggressions on the basis of their accents and their ethnic or religious identities, which often inhibited their sense of belonging. In Harrisonburg, for instance, a 16-year-old girl from Iraq told a story of being on a school bus when she first arrived. In her words: “I didn’t really know English. I couldn’t speak very well and there was [an] American boy [ …] he was talking about my religion in a bad way and talking about me that I’m wearing hijab” (H3.003.1). Several girls remembered peers mocking their religious garments or trying to remove their hijabs.

Although students in both sites almost always described school staff as respectful, educators were not always knowledgeable of students’ cultural backgrounds. This lack of understanding inhibited educators’ ability to reach newcomers in meaningful ways and sometimes led them to commit painful microaggressions against their students. In Austin, a 17-year-old girl from Iraq recounted a time her substitute teacher used her as an example of “cultural change,” as she had supposedly stopped wearing a hijab after arriving to the U.S. The girl was not only offended for having been singled out in class, but also because she had never actually worn a hijab. As she explained, “I found it kinda rude because you can’t just like assume that ‘oh yeah, she took it off and whenever she got here’” (A3.002.1). These stories go well beyond religious garments. Arab boys described being called “terrorists” by their peers and several informants described fights along ethnic lines in their schools, whether between Arabs and Kurds, between MENA students and Latinx students, or within the MENA group.

In addition to the difficulties of adjusting to their new society, families faced ongoing hardships related to their pre-migration and transmigration experiences, including ongoing family separation and mental and psychosocial distress. While most parents did not feel comfortable discussing mental health and psychosocial wellbeing, a few adults and youths described symptoms such as depressive feelings, anxiety, sleeplessness, and aggressive behavior.

While many youths and caregivers continued to struggle with these various challenges, they also described several means by which they had adapted to life in the U.S. in the years following their resettlement. In their efforts to overcome their adversities, girls and boys drew on a rich ecosystem of supports. In keeping with Bioecological Theory’s emphasis on the importance of relationships in child and adolescent development, the remainder of this section describes the various ways in which key actors in an individual’s social ecologies—families, peers, schools, and communities—worked independently and together in both sites to protect newcomers, reinforce their coping strategies, and promote their mental health and psychosocial wellbeing.

### Family

Parents used a variety of strategies to ease their children’s adjustment to life in the U.S. Several took a strict approach to parenting, especially with girls, imposing rigid rules to promote educational achievement and mitigate what they considered social risk. An Iraqi mother in Harrisonburg said of her daughters, for instance, “I advise them to keep only one friend because of conflict or problems” (H2.009.1). Both parents went on to explain that they considered their daughters to be at greater risk of harm than their son and that such advice was a matter of safety and preserving tradition.

Parents described varying degrees of strictness, with some banning or limiting social media use, others restricting social and extra-curricular activities, and still others attempting to act as a gatekeeper between their children and their children’s friends. One Iraqi mother in Austin discouraged her children from becoming friends with other Iraqis struggling to adjust to school. In her words, “I’ve tried to keep my children away from them because, as you know, the good things spread and the bad things also spread” (A2.015.1). Instead, she pushed her children to befriend native English speakers. As she explained, “I always encourage them to fit into the new society, to have communication, under my control and my supervision.” Another Iraqi couple described a painful tension they felt between wanting to become more flexible with their children so as to allow them to fit in with the broader community, and not wanting to lose their children’s respect (H2.010.1 and H2.011.1).

In addition to keeping their children safe and guiding their social development, many parents also considered it their responsibility to maintain their children’s connection with their national or ethnic heritage. As an Iraqi mother in Harrisonburg explained about her children, “we want them to learn about all the traditions [ …] And, and we taught them to speak Arabic, to write Arabic, and to read Arabic” (H2.003.1). A Sudanese mother in Harrisonburg echoed this sentiment, but noted the challenge of this undertaking. In her words, “we speak Sudanese [ …] we try to teach them the culture, Sudanese culture, traditions, but they are refusing everything” (H2.005.1). She went on to explain that “The school already teaches them in a new culture,” making it difficult to keep their Sudanese heritage relevant and exciting for them. She commented that schools could play an important part in encouraging students to maintain and celebrate their heritage.

Parents and older siblings also tried to take an active role in adolescents’ educational lives, with differing degrees of success. Caregivers typically attempted to motivate adolescents by focusing on career objectives, particularly specialized, high-income professions such as medicine and engineering, which were especially difficult to achieve for those newcomers who had missed years of schooling and had arrived at an older age. For some parents, this encouragement was enough, as they felt incapable of engaging with schools meaningfully themselves, and trusted their children to navigate the system on their own. When asked about her son’s college options, for instance, a mother from Iraq responded, “We don’t go into details. We don’t know. He knows better about college” (H2.012.1). In a male FGD in Harrisonburg, several students described a similar parental detachment, explaining that as long as they continued to receive fair grades and did not get into trouble, their parents did not engage much in their educational lives. These students were usually at a disadvantage in school, compared to those with parents who took more active roles in their children’s education. Some newcomer parents, for example, described monitoring their children’s report cards, helping with homework, and keeping in constant contact with teachers, whether by phone, email, mobile phone applications, or routine visits to schools. Parental involvement with schools seems to have been influenced not only by parenting style, but also parental education, English language ability, free time, and student performance.

### Peers

Students took great comfort in one another’s friendship and support. Girls and boys in both cities consistently remembered the peers that welcomed them on their first days of school. Peers oriented newcomers to their new environment and introduced them to their classmates, promoting a sense of belonging. Although schools did not usually institutionalize formal, peer-led orientations, students who had been welcomed when they first arrived often made sure to help new arrivals in turn. As an Iraqi girl in Austin remembered, “I know how lost I was, so I really don’t want other people to be lost [ …] I’m planning to do a thing like with [a recently arrived] family that I’m ‘gonna teach their kids English, help them with homework” (A3.002.1). When possible, newcomers usually made friends with other Arabic-speaking students at first, who interpreted for them in class, taught them some English, and helped them to understand their course schedules and academic and extra-curricular opportunities. Students who did not have other Arabic-speakers in their schools tended to have more initial difficulty adjusting. In Austin, for example, an Iraqi girl remembered being the only Arabic-speaker in her school in the following terms:

*I was like the only Arabic girl and I had recently moved towns. And the town was like most populated with like, Latinos and like Latinas, and so they weren’t very nice that I didn’t speak Spanish. And they would like speak behind my back. And I got like very—I got angry, and sad, and it didn’t, like, do well for me* (Austin, Girl, Iraq, A3.014.1).

Relationships between MENA students and students of Latinx origin tended to be especially tense when the latter constituted a large proportion of the ELL group and drew a majority of the resources and attention from the schools. For the most part, however, MENA students in both cities quickly made friends with non-Arabic-speaking ELLs in their classes. Moreover, as their English improved and they began to take classes with the general student body or to participate in extra-curriculars, many became friends with U.S.-born students as well. For most newcomers, supportive friends were those who were genuinely interested in learning about them and their background, who withheld judgment and laughter when they struggled in class, and who stood up for them when other peers bullied them. An Iraqi girl in Harrisonburg, for instance, recounted a time when her friends defended her against a male classmate who repeatedly followed and harassed her. As she remembered, “when he comes around, they were just yelling at him or trying to fight with him, and he was just leaving after that” (H3.003.1).

### School and community

Schools and communities took numerous measures to support youths and their families. Harrisonburg’s Newcomer Program offers one model of support to ease students’ school adjustment. In this program, newcomers from every background received sheltered instruction through English immersion courses as well as cross-cultural orientation classes until they reached a basic level of language proficiency. In order to promote friendships outside of the newcomer community, students also took some substantive (e.g. math) and elective (e.g. art) courses with the general student population. Teachers and students alike considered the Newcomer Program useful for learning basic English, building a supportive community of peers, and acclimating to the school environment more broadly. As a Sudanese girl remembered, “they put me [in] Newcomer and the teacher [was] always caring and helping and stuff” (H3.004.1). Notwithstanding the program’s considerable strengths, staff still thought it could be improved. One key informant explained that students graduating from the Newcomer Program often still lacked sufficient English proficiency to keep up with students in the more advanced classes at the school. As the informant explained:

*They have the academic chops; they have the motivation; they have the work ethic. Where, if we would scaffold the language appropriately, they could be succeeding like crazy in these courses. But those* [advanced classes] *are the classes where we see the least diversity* (H1.08).

In Austin, resettled families were dispersed around the city, which complicated efforts to coordinate specialized supports across the school district and led to considerable variation in how schools welcomed newcomers. The high school with the highest concentration of refugees did not offer a class designed for refugees or newcomers but, instead, students were enrolled in a standard English as a Second Language (ESL) class, with all other ELLs. The teacher of that class also supported the school’s faculty to meet student need, whether through professional development sessions, adjustment of lesson plans, or coaching.

Austin’s Refugee Family Support Office was central to the city’s ability to respond to the needs of its resettled students and their families. This district office worked across AISD’s departments and schools, as well as several outside organizations, to provide a range of supports. The team, which consisted of a small staff representing the most common refugee language groups, worked closely with schools at every level. Although its central function was to deliver language services, the office also oversaw school registration for refugee students, coordinated tutoring activities, collaborated with resettlement agencies to meet basic needs, collected data on student and family needs, conducted professional development around the district for school faculty and service providers, and informed district policies to improve the context of reception for refugee students. Of course, each of these activities presented its own sets of challenges. With students spread across such a large number of schools, for example, the office struggled to monitor needs throughout the district. As one respondent explained, “there’s a lot of things we don’t even know about until we find out, you know, months later” (A1.03). For their part, school personnel did not always have the time or incentive to attend professional development sessions, making it difficult to diffuse best practices for monitoring and responding to newcomer needs.

Both Austin and Harrisonburg employed liaisons from the MENA community, as well as other immigrant communities. Beyond simple language support, these individuals often tutored and mentored students, ran extra-curricular activities, and helped schools communicate and coordinate with caregivers, including at times visiting parents during their work lunch breaks to discuss their children’s progress. A girl in Harrisonburg described her school liaison in the following terms: “When we came to this country like totally lost, and you don’t know, just don’t know what to do. And she’s just right there for you. She opens the way” (H3.005.1). In Austin, several actors from local universities and NGOs provided additional language and academic tutoring and mentoring for students.

Both cities had recently taken concerted steps towards strengthening SEL in their schools. Harrisonburg had integrated SEL into its curricula and launched programs around restorative justice, cultural competency, trauma-informed care, bullying prevention, and community outreach. In order to provide additional SEL support to newcomers, in particular, Harrisonburg High School launched the Peer Leader Program, together with local partners. In this program, newcomers participated in weekly sessions to share experiences and advice, help welcome new arrivals, and plan activities to engage the broader community. Students participated in a number of additional enrichment opportunities as well. For example, students completed a two-day workshop led by the Center for International Stabilization and Recovery, which, according to one key informant, “focused on how to be a good listener, how to deal with your own stress, how to recognize danger signals in other students” (H1.01). In addition to the Peer Leaders Program, Harrisonburg High School also hosted weekly meetings for Arabic-speaking girls, as well as a monthly Arabic club, all led by the Iraqi school liaison.

At AISD, which reportedly had been implementing SEL in all of its schools as of the 2015–2016 school year, the district was in the process of implementing its SEL 2.0 strategic plan. According to an informant familiar with the plan, SEL 2.0 was an effort to recognize “the centrality of race and equity” and to make “more explicit connections to cultural and linguistic identity” (A1.01). The effort also included elements of restorative practices, mindfulness, and a focus on building adult SEL capacity. Although informants did not share specific plans to tailor this SEL initiative to refugees or the MENA population in particular, members of the Refugee Family Support Office had begun conducting district trainings related to the effort.

In addition to these social and emotional learning measures, AISD, together with local partners, began integrating mental health centers into the school system in 2015, reaching over 40 schools by 2018. The centers took a multi-layered approach to MHPSS, working with several departments to deploy preventive strategies, such as promoting trauma-informed care throughout the schools. The centers also worked with parents and teachers to build referral systems and provided specialized care for students with acute and chronic needs, such as CBT and Trust-Based Relational Intervention. Key informants at the centers acknowledged the particular challenges of working with populations from conflict-affected countries, not just including language barriers, but also differences in conceptualizing mental health and psychosocial wellbeing and stigma around these issues. One informant described her process of trying to destigmatize the issue by referring to specific symptoms, rather than a diagnostic title, and “to bring it back to school and school success,” rather than mental illness and therapy (A1.11). This participant also noted the importance of reinforcing student strengths and helping them manage daily stressors, such as job applications, rather than focusing exclusively on addressing underlying traumas through specialized therapy. This informant recognized the need for greater cross-cultural training and said that the centers were beginning to work with the Refugee Family Support Office on professional development efforts.

## Discussion

Families resettling to the U.S. from conflict-affected countries in the MENA region encounter countless challenges as they adjust to their new society. However, our findings demonstrate that newcomers in Harrisonburg and Austin adapted readily to their new environment and engaged with a rich ecosystem of supports, starting at home and extending through their schools and communities. Guided by Bioecological Theory, our findings demonstrate the critical importance of system-wide support mechanisms that coordinate the efforts of actors at every level to work in tandem, rather than apart [24, 25]. Students received direct educational, mental health, and psychosocial support from numerous actors in their microsystem, including caregivers, peers, educators, and service providers. In addition to working directly with students to reinforce their strengths and address the challenges of adjustment, these actors also worked together in the mesosystem to enhance the overall supportive ecosystem. Teachers, school liaisons, and tutors enabled parents to overcome language and access barriers and to become more involved in their children’s education. Meanwhile, the basic supports from resettlement agencies and other community organizations helped to relieve stressors affecting adult newcomers in the exosystem (e.g. related to employment and finances), allowing them to devote more time and energy to caregiving.

Just as resettled families adapted to their new communities, schools also adapted to their new students, creating a more accommodating context of reception. Specialized personnel with training related to refugees and MHPSS provided coaching and professional development opportunities for teachers and counselors, building more cultural competence and trauma sensitivity. Administrators at the district/division level, for their part, spearheaded initiatives together with community partners and external donors to formalize programs and policies that could support students and families across the school system, whether related to refugees in particular, or SEL and MHPSS more broadly. Such capacity-building efforts have been crucial to a number of previous initiatives to improve school-based MHPSS supports for refugees and asylum-seekers, and several English-language resources have been published in recent years to facilitate professional development [15, 50, 51].

While the study did not test the effectiveness of these various measures for improving student educational performance or mental health and psychosocial wellbeing, it identified several plausible mechanisms through which such improvements could take place. For example, students drew on supportive peer groups to adjust to their new schools, to overcome language barriers and microaggressions, and to gradually create a sense of belonging, despite continued anti-MENA and anti-refugee sentiment in the macrosystem. Recognizing the protective and promotive qualities of such relationships with peers, as well as with family members, educators, and other members of the community, school decisionmakers in these two cities had developed multi-layered supports to build on the strengths of these relationships. By foregrounding SEL and trauma-informed care in the classroom, for instance, AISD aimed to cultivate a more harmonious school climate capable of preventing intergroup conflict, reducing mental health stigma, and encouraging proactive care-seeking through school mental health centers (which themselves provided multiple layers of care). Meanwhile, school liaisons and coordinators worked outside the classroom to bolster parental strengths and further connect them to the school’s support systems. A growing body of evidence supports such integrative, holistic MHPSS approaches that address negative exposures and reinforce strengths across social ecological levels [19, 52, 53]. Examining the linkages between these various actors also exposed fault lines in the two cities’ support systems, however, which may provide useful lessons to guide future interventions and research undertakings and which have significant implications for refugee resettlement policy.

First, both school systems struggled with a fundamental tension between targeted and universal supports, as has been found in previous studies [15]. On the one hand, activities that focused too narrowly on certain subpopulations—whether refugees, Arabic-speakers, or immigrants more broadly—could exacerbate perceived differences between identity groups, undermining newcomer integration and sense of belonging. Without the proper cultural adaptation, on the other hand, universal supports risk either failing to reach newcomers or potentially causing harm [12]. Consistent with the logic of multi-layered MHPSS, actors in both districts attempted to resolve this tension by pairing universal initiatives (e.g. system-wide SEL) with loosely targeted programming for groups with special needs (e.g. Newcomer Program for all recent arrivals) [11]. Youth and parents alike appreciated when program activities were sensitive to their prior experiences and carefully adapted, without singling them out as refugees. Some participants recommended greater reflection of their histories and languages of origin in school and program activities. Such recommendations are supported by a growing body of evidence indicating that newcomers benefit from cultural continuity, though more research is needed in this regard [5].

Second, the complexity of programming and policy efforts, especially in a district as large as Austin, could lead to service gaps, inefficiencies, and miscommunications among actors. Furthermore, the demands on teachers and guidance counselors limited the time they could dedicate to coordination, professional development, and support outside the classroom. School liaisons and AISD’s Refugee Family Support Office emerged as crucial resources, not only for actors within the school system, but also for those in the broader community. School districts/divisions around the U.S. should learn from this coordination model, which our findings suggest depends on stable funding, a dedicated and well-trained staff, and sufficient flexibility to work alongside a broad set of partners.

Finally, despite these various efforts, actors in Austin and Harrisonburg alike had a limited ability to insulate resettled families from the stressors of a macrosystem that is increasingly hostile to refugees in general and those from the MENA region in particular. As described above, students experienced these stressors in their daily interactions, through bullying and microaggressions, despite inclusive school policies. With fewer refugees of MENA origin arriving in the U.S., and reduced budgets for refugee resettlement offices, key nodes in the support networks detailed in this study were already undergoing drastic changes at the time of data collection. Key informants expressed grave concern for what these changes would mean for refugee families, both of MENA origin and otherwise. While these same participants remained confident that, by working together, families, schools, and communities could continue to support newcomers, it is clear that policy interventions at the national and subnational levels will be necessary to sustain and expand an inclusive context of reception [54, 55].

This study had several limitations. By recruiting resettled participants with the help of school districts and community organizations, we were more likely to include families that had received the greatest support. Families which had less contact with these actors (e.g. those whose children had dropped out of school) may have provided greater insight into the limits of existing support efforts. Harrisonburg and Austin were also reputed in their states for being especially welcoming cities. Future studies should investigate supports for resettled families in areas with a less welcoming context of reception, lower ethnic and linguist heterogeneity, and fewer resources. Finally, our study did not include a counterfactual group. Future studies should build on our findings by comparing the outcomes of students resettled from conflict-affected countries in the MENA region against the outcomes of students from other backgrounds.

## Conclusion

In a national political context marked by a growing normalization of anti-refugee sentiment and decreasing political and financial support for refugee resettlement, this study suggests that, by working together, families, peers, schools, and local communities can nevertheless create a welcoming and caring environment that many newcomers in this study considered central to their adaptation and sense of belonging. Although resettled students and families in the study sites struggled with overwhelming pre-migration, transmigration, and post-migration challenges, they also demonstrated a remarkable commitment to seizing the opportunities of their new society, investing in their educational and career advancement, participating actively in their communities, and helping one another. While this study underscores the resilience of newcomers and the value of local support systems, it also reflects the importance of government investment in schools, mental health systems, and resettlement programs that are necessarily for enabling newcomers to achieve their full potential.

## Supplementary information

**Additional file 1.** Summary of Codebook.

## Data Availability

The data that support the findings of this study are available upon request.

## Footnotes

1 Including Algeria, Bahrain, Comoros Islands, Djibouti, Egypt, Iran, Iraq, Israel, Jordan, Kuwait, Lebanon, Libya, Mauritania, Morocco, Oman, Palestine, Qatar, Saudi Arabia, Sudan, Syria, Tunisia, United Arab Emirates, and Yemen.
