# Supplementary material for: Ecologies of care: mental health and psychosocial support for war-affected youth in the U.S
Source: Confl Health. 2019 Oct 21;13:47. doi: 10.1186/s13031-019-0233-x (PMC6802323; doi:10.1186/s13031-019-0233-x)
Supplement: Supplementary file 1 — Additional file 1. Summary of Codebook. [file 13031_2019_233_MOESM1_ESM.docx]

| **Annex 1.** Summary of codebook | | | |
| --- | --- | --- | --- |
| **Theme** | **Code** | **Theme** | **Code** |
| Life experiences and challenges | - Mental health issues - Language challenges - Bullying or fighting - Discrimination - "Bad" influences - Within-group tensions - Inter-group tensions - Pre-flight experiences - Displacement experiences | Intercultural factors | - Acculturation strategies - Reactions to American life and culture - Perceptions of refugees - Religion - Comparison between Arab and non-Arab migrants - Negotiating personal identities - Perceptions of MHPSS |
| School-specific | - School comparisons - School registration, accreditation, and grade placement - Adjustment to new school - Sense of school belonging - School cultural and trauma competence - Potentially harmful practices | Structural  factors | - Political influences - Policies - Gender system - Employment and household finances |
| Linkages | - Partnership - Program coordination - Referrals - Funding | Innovations | - Ideas and recommendations - New approaches - Technology - Data and evaluations |
| Support | - Language support - Academic and career support - Resettlement and basic needs support - SEL support - Community support - Parental support - Support for parents - Peer support - Layer III (Non-specialized mental health support) - Layer IV (Specialized mental health support) - Staff professional development - Reinforcing strengths - Definition of support - Reactions to MHPSS services - Universal vs. targeted supports - Coping mechanisms | Relationships | - Parent-school relationship - Parent-child relationship - Parent-community relationship - School-child relationship - Provider-client relationship - Peer relationship |
